# Observation selection bias in contact prediction and its implications for structural bioinformatics

**DOI:** 10.1038/srep36679

**Published:** 2016-11-18

**Authors:** G. Orlando, D. Raimondi, W. F. Vranken

**Affiliations:** 1 Interuniversity Institute of Bioinformatics in Brussels, ULB-VUB, La Plaine Campus, Triomflaan, Belgium; 2 Structural Biology Brussels, Vrije Universiteit Brussel, Pleinlaan 2, Belgium; 3 Structural Biology Research Center, VIB, 1050 Brussels, Belgium

## Abstract

Next Generation Sequencing is dramatically increasing the number of known protein sequences, with related experimentally determined protein structures lagging behind. Structural bioinformatics is attempting to close this gap by developing approaches that predict structure-level characteristics for uncharacterized protein sequences, with most of the developed methods relying heavily on evolutionary information collected from homologous sequences. Here we show that there is a substantial observational selection bias in this approach: the predictions are validated on proteins with known structures from the PDB, but exactly for those proteins significantly more homologs are available compared to less studied sequences randomly extracted from Uniprot. Structural bioinformatics methods that were developed this way are thus likely to have over-estimated performances; we demonstrate this for two contact prediction methods, where performances drop up to 60% when taking into account a more realistic amount of evolutionary information. We provide a bias-free dataset for the validation for contact prediction methods called NOUMENON.

Next Generation Sequencing technology is providing an unprecedented amount of uncharacterized protein sequences, leading to an exponential growth of sequence databases such as Uniprot^1^. These new sequences provide an indisputable amount of information, and although their amino acid sequence implicitly encodes protein structure and function, a considerable effort is required to explicitly describe what happens at the proteins’ atomic level. Structural biology has contributed enormously in understanding the nature and the properties of proteins, but despite the noticeable technical improvements^2^,^3^,^4^ the experiments remain complex and are not very amenable to large scale *omics* approaches.

Computationally, bioinformatics has risen to this challenge by developing tools to predict missing structural annotations for protein sequences where experimental data is lacking. An enormous number of bioinformatics softwares have been developed with the aim of predicting, for example, secondary structure^5^,^6^,^7^,^8^, solvent accessibility^9^, various post translational modifications^10^,^11^, disordered regions^12^,^13^, backbone dynamics^14^, disulphide bonds^15^,^16^,^17^, protein-protein interactions^18^,^19^ and, importantly, the entire protein structure^20^,^21^,^22^,^23^,^24^,^25^,^26^,^27^,^28^.

Many of these methods use evolutionary information as a powerful resource to improve the reliability of their predictions. This information is collected in the form of Multiple Sequence Alignments (MSAs) using tools such as BLAST^29^ or jackHmmer^30^ and, starting from the late 90s/early 00s, this aspect has become an essential part of most prediction methods^5^,^7^,^8^,^17^,^31^,^32^,^33^. The success of including evolutionary information resides in natural selection, with the protein sequence-to-structure relationship (first suggested by Anfinsen^34^) acting over evolutionary timescales. This leads to a sequence conservation signal of structurally and functionally relevant parts of proteins emerging across related species^26^,^35^,^36^. This effect is strong, with some structural bioinformatics tools showing a clear correlation between the number of homologous sequences retrieved by the alignment algorithm and the reliability of their predictions^15^,^25^,^37^,^38^. Fields in which this effect has been observed include, but are not limited to, functional characterization of linear motifs^39^, domain boundaries identification^40^, DNA-binding sites prediction^41^, disulfide bonds connectivity prediction^15^, fold recognition^42^ and Contact Prediction^25^,^38^.

All bioinformatics tools developed to address protein structure-related tasks share the same, crucial, characteristic: they need a validation procedure based on experimentally determined data to evaluate their performances. The underlying assumption is that if a method works well for the proteins in the validation set, it will also work for ones with unknown structure. In other words, this procedure is reliable only if the validation data is representative of the entire population of protein sequences, with no significant difference between the subset of experimentally investigated proteins and all non-investigated ones. The intrinsic nature of this structure-based validation in structural bioinformatics could be a major cause for *observation selection bias*, where particular properties of an object are correlated with its probability to be sampled.

In this work we show that observation selection bias can indeed skew the performance of structural bioinformatics methods. First, we show a striking difference between the availability of homologs for proteins with a PDB structure and for proteins where only the Uniprot sequence is available, which translates to lower overall NEFF scores^43^, a score equal to the average number of different amino acids in each column of the MSA, and lower average residue entropies for the latter sequences. The performance of structural bioinformatics methods that (i) are trained on experimental structural data and (ii) use evolutionary information to improve their prediction is therefore likely over-estimated with respect to real case applications. We show that this is indeed the case in the Contact Prediction (CP) field, where protein structures are predicted by inferring inter-residue contacts. The CP field fits criteria (i) and (ii), with a well documented correlation between the number of homologous sequences available and the prediction performances, so making the observation selection bias immediately and directly relevant^25^,^37^,^38^. Moreover, the widely adopted use of unsupervised prediction methods in this field facilitates the fair evaluation of the prediction in function of different datasets, without the confounding overfitting effects of supervised methods. Based on NOUMENON, a new CP dataset containing 150 proteins where the 3D structure observation selection bias is removed by emulating a more realistic homologue sampling, we show that CP performances drop dramatically (see Fig. 1 for an overview of our analysis). NOUMENON is available to the community for the development of future tools. Overall, our findings question the de facto applicability of structural bioinformatics tools that fit the two criteria on real cases, *i.e.* structurally undetermined proteins with a representative set of homologs, and calls for a more careful evaluation of their performances. This is essential not only to understand the reliability of the results, but also to avoid long-term negative effects on structural bioinformatics research: the necessity to boost the performances of a tool in order to achieve a publication could lead to a positive selection of methods that take advantage from information that is not available in real case applications.

## Results and Discussion

### Investigating the relationship between retrieved homologous sequences and the availability of 3D structures

**Figure 1:**
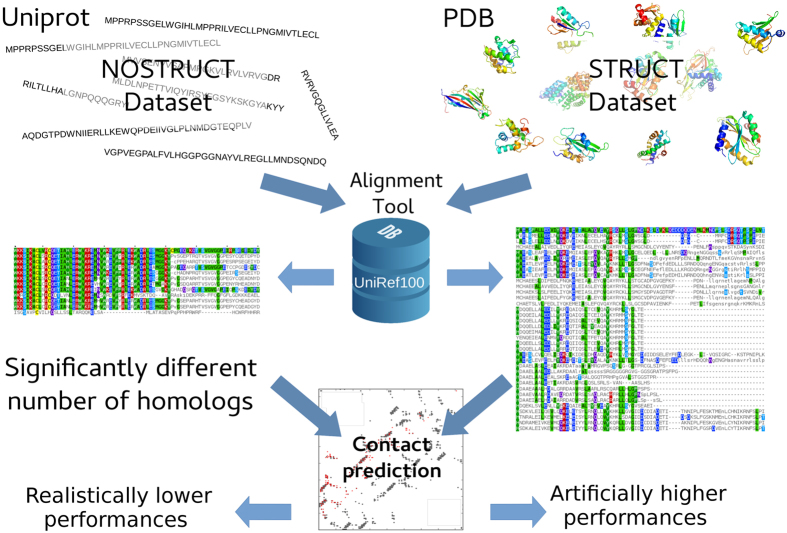
Overview of the analysis. There is a significant difference in the number of homologs that can be retrieved for a protein with and without a solved structure. This can lead to an overestimation of the performances of methods that use this kind of information, as we show for contact prediction, where this effect is very strong.

We first evaluated the amount of homologous sequences that can be retrieved for proteins with known or unknown three dimensional structure. From Uniprot20^44^ we created NOSTRUCT, a dataset of 5000 randomly selected non redundant, experimentally verified sequences containing no homology with proteins that have an experimentally determined structure in the PDB (see Fig. 1 for an overview and Methods for details). We then used NOSTRUCT to infer the distributions of available homologs for proteins without a PDB entry, which are the real case applications of structural bioinformatics methods. We also created the STRUCT dataset of non redundant sequences from PDB, where we retrieved all the sequences in the PDB and clustered them to remove proteins sharing more than 20% sequence similarity. From the resulting set of 16476 proteins we randomly selected 5000 sequences.

**Figure 2:**
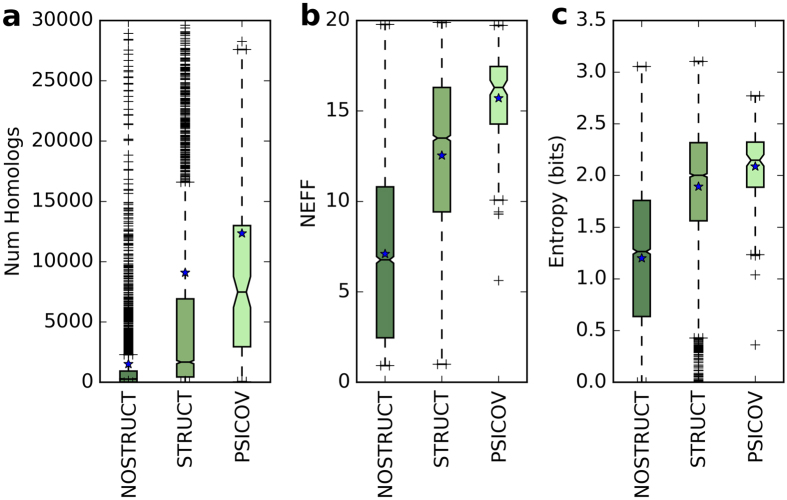
(**a**) Distributions of the number of homologous sequences retrieved by jackhmmer (with 1 iteration and E-value = 0.0001) for NOSTRUCT, STRUCT and PSICOV datasets. (**b**) Distributions of the NEFF scores calculated on the homologs retrieved by jackhmmer for NOSTRUCT, STRUCT and PSICOV datasets. (**c**) Distributions of the average entropy for the alignments in the three datasets.

We then retrieved the homologous sequences for the members of the NOSTRUCT and STRUCT set using jackHmmer^30^, one of the most used tools for homology retrieval and alignment in structural bioinformatics in general and CP in particular^15^,^22^,^25^,^38^. The distribution of the number of retrieved homologous sequences (Fig. 2a) shows that the difference between the distributions for these sets is so significant that the average number of homologs in the STRUCT dataset is about 6 times larger than in NOSTRUCT. The two-tailed Wilcoxon ranksums test gives p-values smaller than 10^−300^ and allows us to state that the number of retrievable homologous sequences is highly correlated with the protein having a solved structure in the PDB or not. Figure 2a also shows the distribution of the retrieved homologs for the 150 proteins in PSICOV dataset^22^, which is commonly used in CP. The number of homologs available in PSICOV is even greater than in STRUCT (ranksums p-value = 5.78 × 10^−17^) and definitely not comparable with NOSTRUCT (p-value = 2.28 × 10^−66^). While it is well known that the sequences in the PSICOV dataset tend to have more homologs, our results show that this difference is more fundamental and concerns a discrepancy in homologs between proteins from Uniprot and proteins with a solved structure in the PDB. This difference affects every dataset based on a random selection of protein structures. We performed the same analysis on the dataset used for the Critical Assessment of Structure Prediction (CASP11)^45^. The results are shown in Supplementary Figure 1. While the number of available homologs is much lower than in STRUCT dataset, it is still significantly higher (evalue = 3.28 × 10^−6^ for the number of homologs, evalue = 5.71 × 10^−15^ for the NEFF) than in NOSTRUCT.

To ensure that this effect is not due an uncontrolled variable that affects the capability of the alignment tools to retrieve homologous sequences, we investigated several factors. First, the number of homologs is only poorly correlated with protein length (Pearson’s *r* = 0.16) and the contacts density (the number of contacts in a protein divided by its length) (*r* = 0.06) (see Supplementary Figures 2 and 3). A more biophysical reason could be that the alignment algorithms are less able to deal with fully or partially disordered proteins, which are also difficult to study with structural biology methods (such as X-ray diffraction) and would therefore be much less represented in the STRUCT dataset. We ran a single-sequence disorder predictor (IUpred^46^) on the NOSTRUCT dataset, and found there is only a very low correlation (Pearson’s *r* = −0.06) between the percentage of predicted disordered residues for a protein and the number of homologs that are retrieved, asserting that protein disorder does not significantly affect our results (see Supplementary Figures 5 and 6).

Finally, we also evaluated if a different distribution of the organisms from which the proteins originate could influence the number of homologs in the STRUCT and NOSTRUCT databases. The NOSTRUCT dataset has the same distribution of organisms as observed in the experimentally verified Uniprot sequences, while the PDB contains a much higher fraction of bacterial proteins. To verify if a simple organism-based filter could remove all possible biases, we replicated the analysis shown in Fig. 2a with a stratification per taxonomic domain: Supplementary Figure 7 shows that the distribution of the homologs between STRUCT and NOSTRUCT are different even when considering each taxonomic domain independently.

These results are striking, but the number of available sequences may not be the best criterium for evaluating the difference between the datasets, as alignment methods may retrieve very similar sequences and provide a redundant collection of homologs. A higher number of homologs would then not necessarily correspond to a higher information content. We also calculated the NEFF score, which relates the average sequence variation within each MSA, and ranges from 1, if all the sequences are identical, to 20, if there is complete variability in every column. The NEFF score distribution (Fig. 2b) shows that, in comparison to Fig. 2a, proteins with structures in the PDB not only tend to have more known homologs, but the information content of their MSAs tends to be higher: the median NEFF for the STRUCT dataset is twice the median for NOSTRUCT and the ranksums (p-value is lower than 10^−300^). Again, the PSICOV dataset has a higher median NEFF, highlighting a striking difference with both STRUCT (p-value = 5.6 × 10^−18^) and NOSTRUCT (p-value = 1.52 × 10^−73^).

Finally, Fig. 2c shows the distribution per dataset of the averages of the per-residue entropies over each sequence. The PSICOV dataset has the higher average information content (the median is 2.15 bits) and it is significantly higher then both STRUCT (ranksums p-value = 0.00016) and NOSTRUCT (ranksums p-value = 4.6 × 10^−49^). NOSTRUCT has a median entropy of 1.26 bits and is in turn significantly different than STRUCT (p-value < 10^−300^). More details are available in Supplementary Figures 8 and 10.

### The relevance of the homologs availability in Structural Bioinformatics: the Contact Prediction case

**Figure 3:**
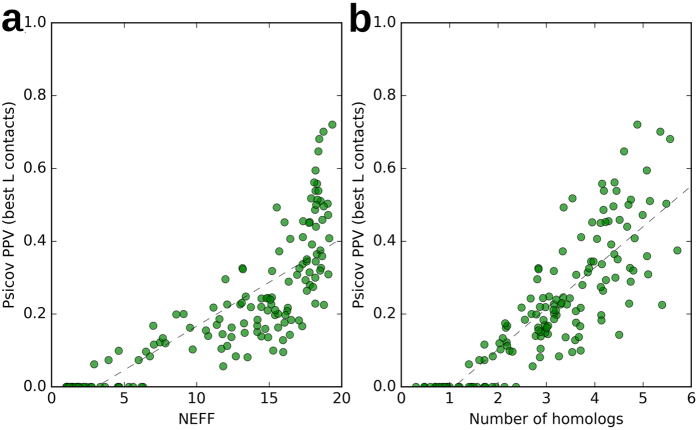
(**a**) Shows the correlation between the NEFF and the PSICOV performances on 150 proteins sampled from the STRUCT dataset (Pearson’s correlation coefficient is 0.83). (**b**) Shows the correlation between the number of homologs (expressed in thousands of homologs) and PSICOV performances on the same proteins (Pearson’s correlation coefficient is 0.70).

The relevance of the availability and quality of MSAs for prediction performances in structural bioinformatics is well documented^5^,^7^,^8^,^15^,^17^,^31^,^32^,^33^ and it is particularly evident in CP, both in terms of the number of available homologs^38^ and of information content (NEFF)^25^. Figure 3a shows the correlation between the NEFF of the MSAs and PSICOV performances (*r* = 0.83) and Fig. 3b shows the correlation between the number of homologs and PSICOV performances (*r* = 0.70) on 150 proteins sampled from the STRUCT dataset. This confirms the previously determined correlation between available evolutionary information and CP performances for plmDCA, PSICOV, PconsC and PconsC2 on the PSICOV dataset^25^.

These results question the consistency of the accuracy that CP methods claim, since their published performances are calculated on protein datasets that are significantly enriched in number of available homologs compared to real application cases.

### NOUMENON: a new CP dataset with homologous distribution similar to real application cases

To test how much the predictive ability of CP methods are influenced by the scarcity of homologs observed for most proteins, we devised a new CP dataset, called NOUMENON. We designed it to provide a benchmark for CP methods free from the observation selection bias due to the correlation between number of homologs and availability of PDB structures: proteins in NOUMENON have been selected in order to match the same distribution of homologs observed in NOSTRUCT dataset.

From STRUCT we sampled a set of 150 non-redundant proteins ensuring that the distribution of their homologs (obtained with 1 iteration of jackhmmer) was as close as possible to the randomly determined Uniprot distribution in NOSTRUCT (see Methods for details). Supplementary Figure 9 shows a comparison between the homologs distribution in NOSTRUCT and NOUMENON.

**Figure 4:**
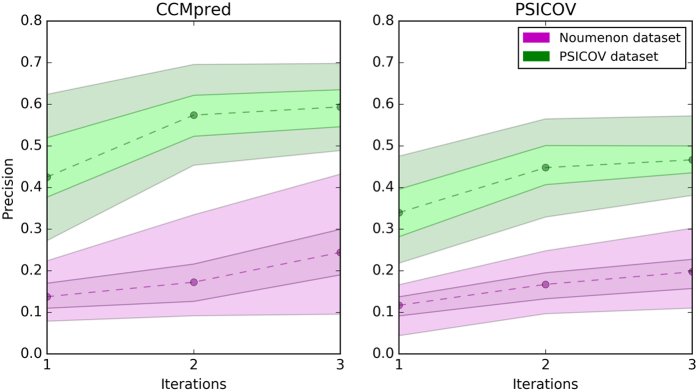
Plots showing the medians of the performances of CCMpred and PSICOV on NOUMENON dataset (magenta) and PSICOV dataset (green). The shaded area indicates for each iteration the data between the 40th and the 60th percentile and between the 25th and 75th percentile.

We then tested the PSICOV^22^ and CCMpred^24^ unsupervised contact prediction methods on the NOUMENON dataset and compared the results to the ones obtained on the widely adopted PSICOV dataset. We selected PSICOV because it is a landmark method in this field and CCMpred because is the most recent implementation of a popular statistical mechanics based method^23^. Figure 4 shows the median precision scores (PPV) for the best L predicted contacts with sequence separation greater than 4 residues, where L is equal to the sequence length of each protein (see also Supplementary Table 1 for the mean precisions). Both PSICOV and CCMpred generally experience a 50–60% drop in performance when tested on NOUMENON. The performance, as expected, improves when increasing the number of iterations for jackhmmer, meaning more homologs are collected.

To show that this dramatic drop of the performances is a genuine over-estimation of the performances and not due to confounding effects hidden in the different nature of the protein structures selected in NOUMENON and PSICOV datasets, we took the best performing alignments, obtained with 3 iterations of jackhammer and ran an additional experiment in which we artificially cut the sizes of the MSAs collected for PSICOV dataset in order to match the number of homologs available for NOUMENON. We then computed the performances of PSICOV and CCMpred predictors on this version of PSICOV dataset with these artificially reduced number of homologs: PSICOV yielded to a best L mean precision of 0.20 and CCMpred of 0.27 (see Supplementary Table 1). Artificially reducing the number of homologs on PSICOV dataset thus gives 7–9% lower average scores than the predictions with the same number of homologs obtained on NOUMENON. This indicates that NOUMENON does not penalize the scores of these predictions more than what is expected solely due to the reduced number of homologs available.

## Conclusions

Many structural bioinformatics methods that predict structural characteristics from protein sequence validate their performance on known protein structures and use evolutionary information in to boost prediction performance. We show here that proteins for which experimentally determined structures from the PDB exist have significantly more homologous sequences available, with a higher information content in the corresponding MSAs, than typical proteins from Uniprot without characterised structures. This represents an observation selection bias that inflates prediction performance because more homologs are available for exactly those proteins that constitute the validation sets: the evolutionary information available for validated proteins differ from the real case applications for which bioinformatics methods intend to provide useful annotations.

We demonstrate this observation selection bias with contact prediction (CP) methods, for which the dependence between performances and number of homologs is particularly pronounced; the datasets used for the validation of CP methods are even more enriched with homologs in comparison to the general distribution of homologs found in the PDB. In order to properly assess the performance of CP methods on real case applications, the homolog distributions have to reflect the general situation found in Uniprot. The NOUMENON dataset we introduce here addresses the observation selection bias for CP methods, and shows that the realistic performance of the methods is 50–60% lower than reported. We hope developers of future CP methods will validate their softwares on NOUMENON, or similar datasets, so the effective performance of their tools is assessed.

The reason for this bias is difficult to pinpoint and likely stems from several causes. We hypothesise that it mainly results from the focus of structural biology on proteins for which there is a clear medical or biological interest. In order to motivate the significant investment of time and resources required for an experimental study, there must already be a disproportionate amount of information available, such as known similar proteins or a connection to disease. This effect leads to a non-homogeneous distribution of information among the proteome.

How much other structural bioinformatics methods are affected by the number of available homologous sequences is more difficult to determine because many approaches are based on datasets where particular sequences are directly related to the per-residue information to be predicted (e.g. secondary structure), often with supervised machine learning approaches. This way more general principles can be extracted from the training set, but there is likely still an effect of the number of homologous sequences given the increase in performance evolutionary information can provide^7^,^8^,^41^,^42^.

Directly showing the extent of the observational selection bias effects within every possible subfield of structural bioinformatics is beyond the scope of this paper but, as attested by Fig. 2c, the average over the sequence of the per-residue sequence entropies shows that PDB-related datasets such as PSICOV and STRUCT have a much higher information content from the pure information theory point of view. This implies that, when training or validating methods on PDB-related datasets, more information is available to the bioinformatics tools using sequence profiles or position-specific scoring matrices (PSSMs) with respect to the information available for uncharacterized Uniprot sequences. Supervised learning approaches that use this evolutionary information will therefore be trained and validated in conditions of significantly higher levels of information than expected in the real-use cases, undermining their general applicability and the reliability of their predictions.

Since the ultimate goal of structural bioinformatics tools is to provide *in silico* annotations for poorly characterized protein sequences without experimentally determined information, the inherent observation selection bias we demonstrate here should be taken into account. It may have long-term effects on the evolution of structural bioinformatics as a field: the usage of evolutionary information can drastically boost the performances of some methods, but also increases the distance between proteins in the validation set and the large share of poorly annotated proteins that exist in nature. The risk is that in order to push the performances of newly developed tools, authors often extract as much information as possible from MSAs, making them even more dependent on this – still relatively scarce – type of data. This leads to a unjustified positive selection of methods that use evolutionary information: tools that are less dependent on the number of homologs and that could be more suitable for real application cases may remain unused or even unpublished because their apparent performance is not as good as the other methods, notwithstanding their wider applicability. In addition, other possible causes of an observation bias effect for structural bioinformatics methods based on the PDB, such as for example the high proportion of bacterial sequences, should be taken into account. Further developments in this exciting field of science can only benefit from a better and closer look at the datasets that underly the wide range of different flavours of prediction methods.

## Methods

In the following section we describe in detail the procedure followed to obtain the results shown.

### Building the NOSTRUCT dataset

The NOSTRUCT dataset was built starting from the June 2015 version of Uniprot20^44^, a clusterized version of Uniprot available at http://wwwuser.gwdg.de/compbiol/data/hhsuite/databases/hhsuite_dbs/. It contains Uniprot^1^ sequences organized in similarity-based clusters of proteins where the inter-cluster sequence identity is lower then 20%. From each cluster we extracted at most a single sequence with experimental validation at the transcriptome or proteome level (using the UniProtKB^47^ nomenclature) and with a sequence length between 50 and 1500 residues, selecting a total of 268730 proteins. This length threshold removes less then 3.5% of uniprot sequences, while making the analysis of the proteins computationally feasible. In order to keep only proteins with no evolutionary relationship with proteins that have structures in the PDB, we ran a BLAST^29^ search against the PDB^48^ database for each selected sequence. We considered only proteins for which BLAST returned no hits with E-value = 10^−7^ as threshold. In this way, if no match is found, we can assert that the protein has no close homologous with sequences in PDB, and can thus be considered a possible real case application for structural bioinformatics tools. We stopped the run as soon we found 5000 proteins with no relation to known structures. These sequences constitute the final NOSTRUCT dataset.

### Building the STRUCT dataset

We extracted all the protein sequences from PDB database with resolution lower then 2Å and we applied the same length filter used for NOSTRUCT, keeping only proteins with lengths between 50 and 1500 residues, obtaining a total of 47423 sequences. We then clusterized these proteins using BLASTCLUST^29^ with 20% sequence identity at 90% coverage, obtaining 16476 clusters. In order to remove redundant sequences, we randomly selected 5000 clusters from which we extracted a single protein from each. These 5000 proteins constitute the final STRUCT dataset.

### Multiple Sequence Alignments

The multiple sequence alignments (MSAs) in this study have been obtained using jackhmmer^30^. We chose this tool because it is widely used in Bioinformatics and in particular in Contact Prediction field^24^,^25^,^37^. All the alignments in this work have been computed searching homologs in the 2015 version of Uniref100 (ftp.uniprot.org/pub/databases/uniprot/uniref/uniref100).

Jackhmmer can perform iterative search for homologs, but we used a single iteration search to build the distributions shown in Fig. 2 because (i) the large number of sequences in NOSTRUCT and STRUCT required a relatively fast approach and (ii) we assume the number of homologs for each protein to be a monotonic function of the iterations. In other words, if a protein *P* has *x*_*i*_ homologs at iteration *i*, it will have *x*_*i*+*k*_ ≥ *x*_*i*_ homologs after *i* + *k* iterations. Supplementary Figure 4 shows that this assumption holds in the vast majority of the cases we sampled and that sequences with a small number of homologous retrieved at iteration *i* do not benefit from larger amounts of iterations; namely, the proteins with fewer homologs at 1 iterations are also the ones with fewer homologs at 5 iterations. Our results for 1 iteration are therefore also relevant for multiple iterations that introduce more depth in the MSA.

### Building NOUMENON

The NOUMENON dataset was built by sampling 150 proteins from the STRUCT dataset. The sampling has been constrained in order to preserve the distribution of the number of homologs observed in NOSTRUCT, obtaining a validation dataset for CP methods free from the bias due to the correlation between PDB structures and number of homologs (shown in Fig. 2a). For the extraction of the *real* contacts, we adopted the same contact definition used in CASP: we consider two residues to be in contact when their C-*β* are closer than 8 Ångstroms (C-*α* for glycines).

To make NOUMENON even more suitable for the development and validation of CP methods we applied additional filters, traditionally used in CP literature^22^,^25^. In particular, we ensured that all the proteins in NOUMENON have (i) at least *L* contacts (with *L* equal to the length of the protein) and (ii) a length comprised between 50 and at 275 residues (as in PSICOV dataset^22^). Supplementary Figures 2 and 3 show respectively that there is a poor correlation between the protein lengths or the number of real contacts with the number of retrieved homologous. From these plots we can conclude that these filtering do not introduce other observational selection biases.

The NOUMENON dataset is available at http://ibsquare.be/noumenon.

## Additional Information

**How to cite this article**: Orlando, G. *et al*. Observation selection bias in contact prediction and its implications for structural bioinformatics. *Sci. Rep.* **6**, 36679; doi: 10.1038/srep36679 (2016).

**Publisher’s note:** Springer Nature remains neutral with regard to jurisdictional claims in published maps and institutional affiliations.

## Supplementary Material

Supplementary Information
