# Supplementary material for: Observation selection bias in contact prediction and its implications for structural bioinformatics
Source: Sci Rep. 2016 Nov 18;6:36679. doi: 10.1038/srep36679 (PMC5114557; doi:10.1038/srep36679)
Supplement: Supplementary Information [file srep36679-s1.pdf]

Observation selection bias in contact prediction and its implications for structural bioinformatics

G. Orlando[1,2,3,†], D. Raimondi[1,2,3,†], W.F. Vranken[1,2,3,*]

1 Interuniversity Institute of Bioinformatics in Brussels, ULB-VUB, La Plaine Campus, Triomflaan

2 Structural Biology Brussels, Vrije Universiteit Brussel, Pleinlaan 2

3 Structural Biology Research Center, VIB,1050 Brussels, Belgium

† Contributed equally

*Corresponding Author: wvranken@vub.ac.be

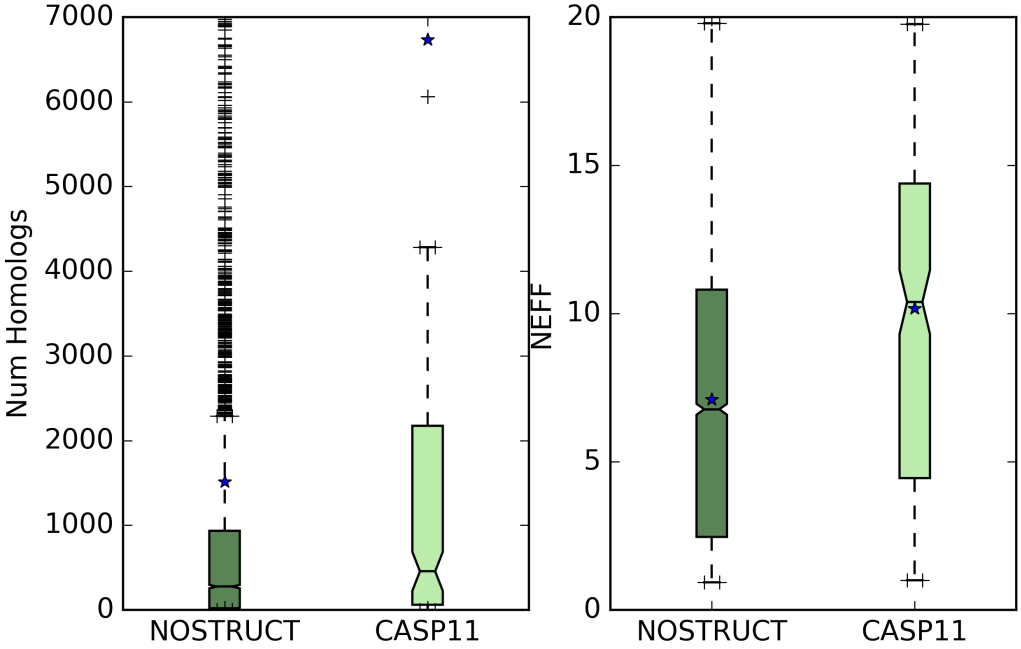

Figure 1: *Boxplots showing the difference in the number of homologs and NEFF between the NOSTRUCT and CASP11 datasets.*

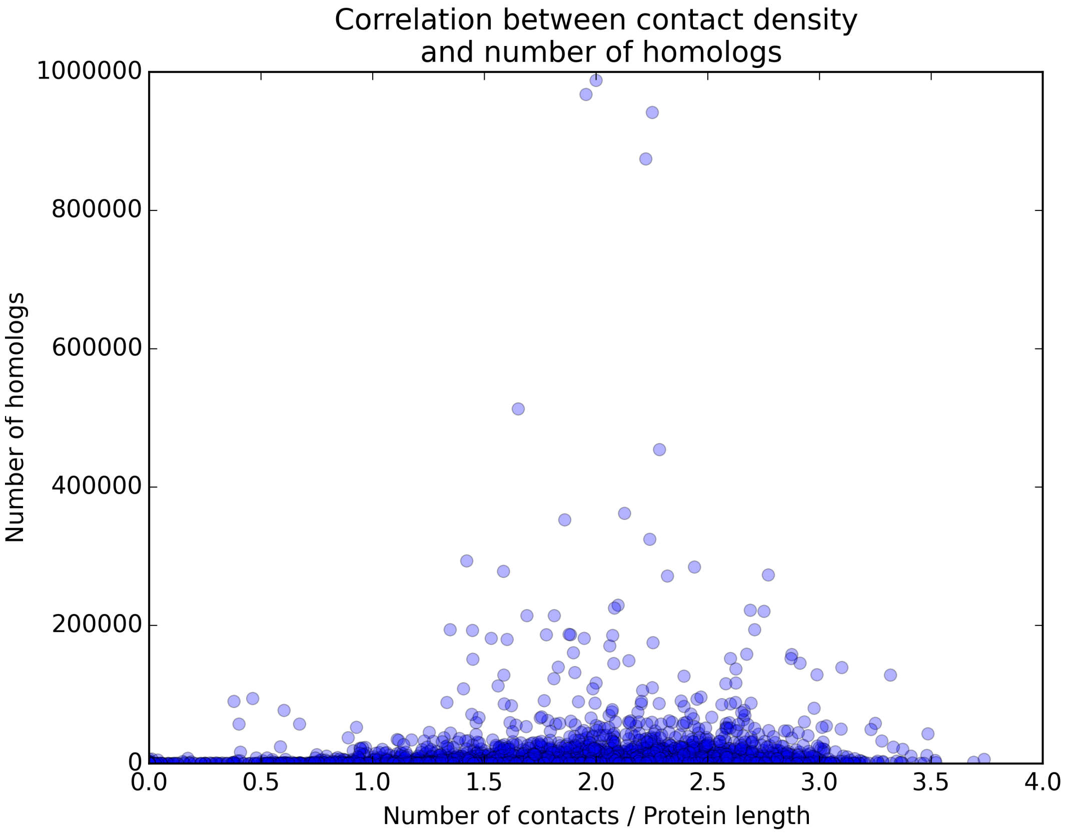

Figure 2: *Scatter plot showing the correlation between the contact density within each protein chain and the number of available homologs. Pearson correlation is* $r = 0.06$*.*

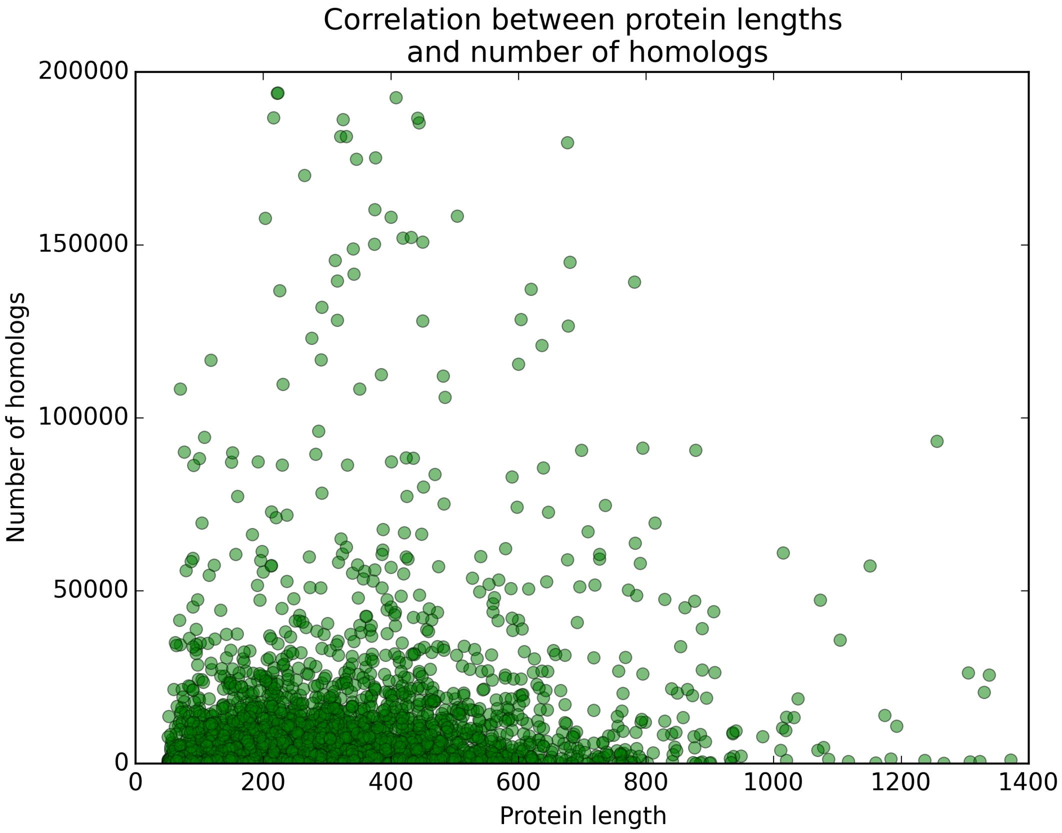

Figure 3: *Scatter plot showing the correlation between the number of retrieved homologs and the length of the protein. Pearson correlation is* $r = 0.16$.

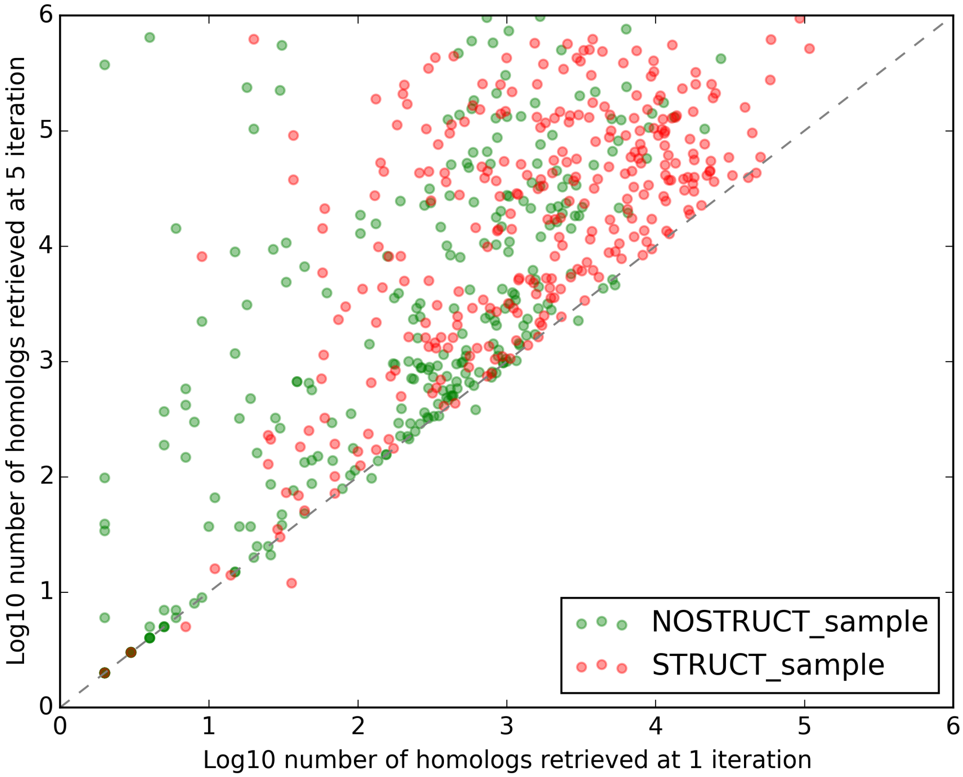

Figure 4: *Relationship between the number of homologs retrieved with a single iteration of JackHMMer and the ones retrieved with 5 iterations. From this plot it is possible to see that in the nearly all the cases increasing the number of iterations leads to more retrieved homologs.*

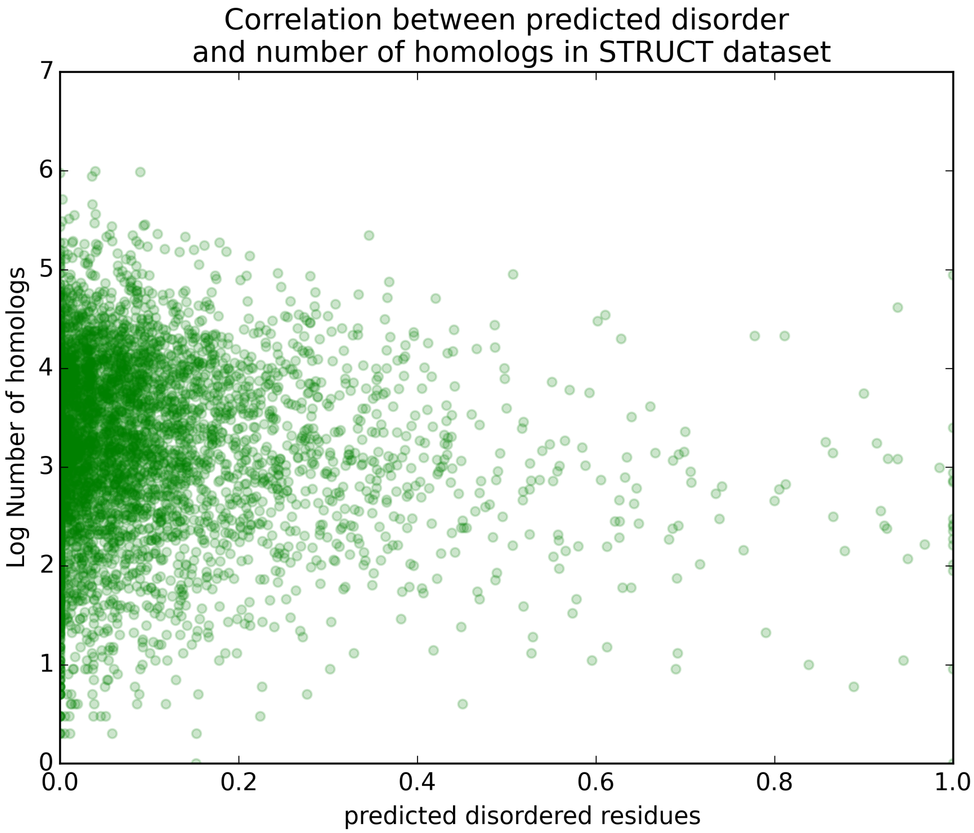

Figure 5: *Scatter plot showing the correlation between the fraction of disordered residues within each protein chain of the STRUCT dataset as predicted by IUPRED. Pearson correlation is* $r = -0.035$.

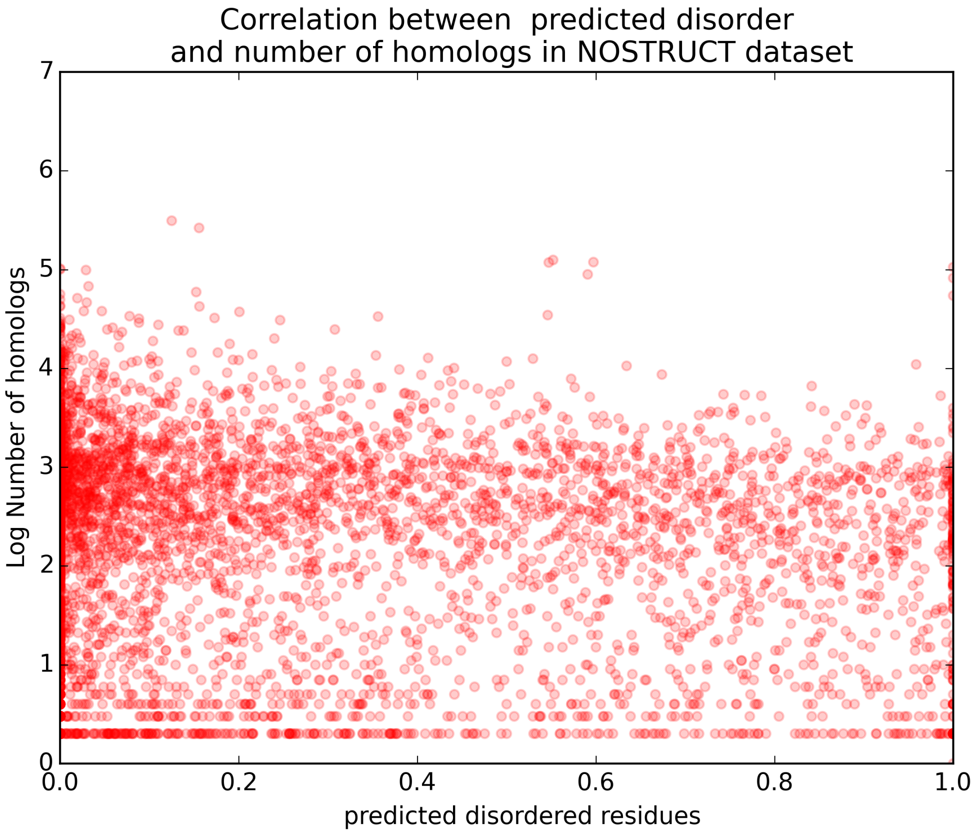

Figure 6: *Scatter plot showing the correlation between the fraction of disordered residues within each protein chain of the NOSTRUCT dataset as predicted by IUPRED. Pearson correlation is* $r = -0.06$.

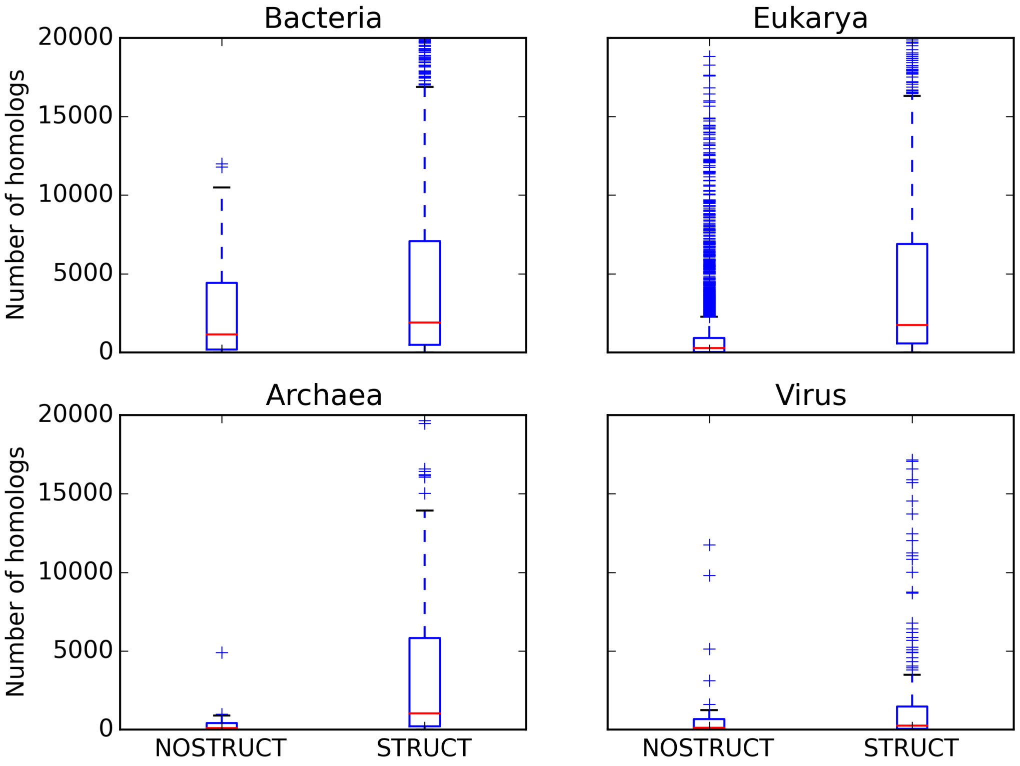

Figure 7: *Boxplots showing the number of homologs retrieved for the STRUCT and NOSTRUCT datasets stratified by organism using the taxonomic domain, the highest taxonomic rank.*

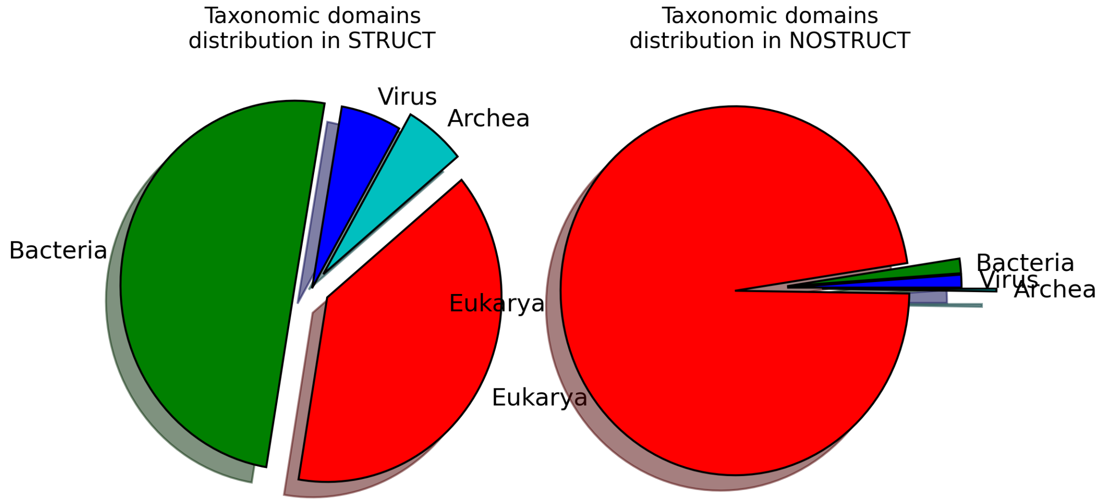

Figure 8: *Pie charts showing the organism composition, in terms of taxonomic domain, of the STRUCT and NOSTRUCT datasets. Half of the proteins in STRUCT are from Bacteria and 38% are from Eukarya. These percentages are very different in NOSTRUCT, which is highly enriched in Eukarya (97%). These pie charts are representative of the organism distributions observed in PDB and SwissProt respectively.*

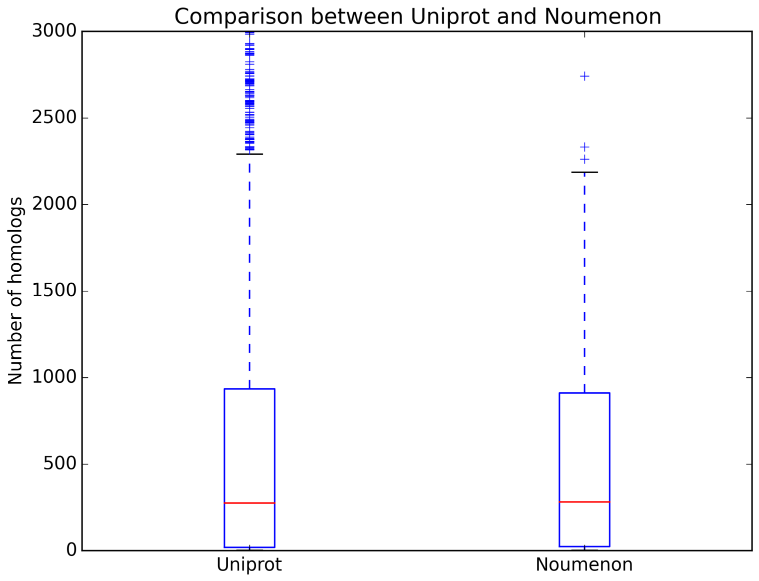

Figure 9: *Distributions of available homologs for the NOSTRUCT and NOUMENON datasets. The two tailed Wilcoxon ranksum p-value is 0.56 and thus the null hypothesis that the two sets of measurements are drawn from the same distribution can not be rejected.*

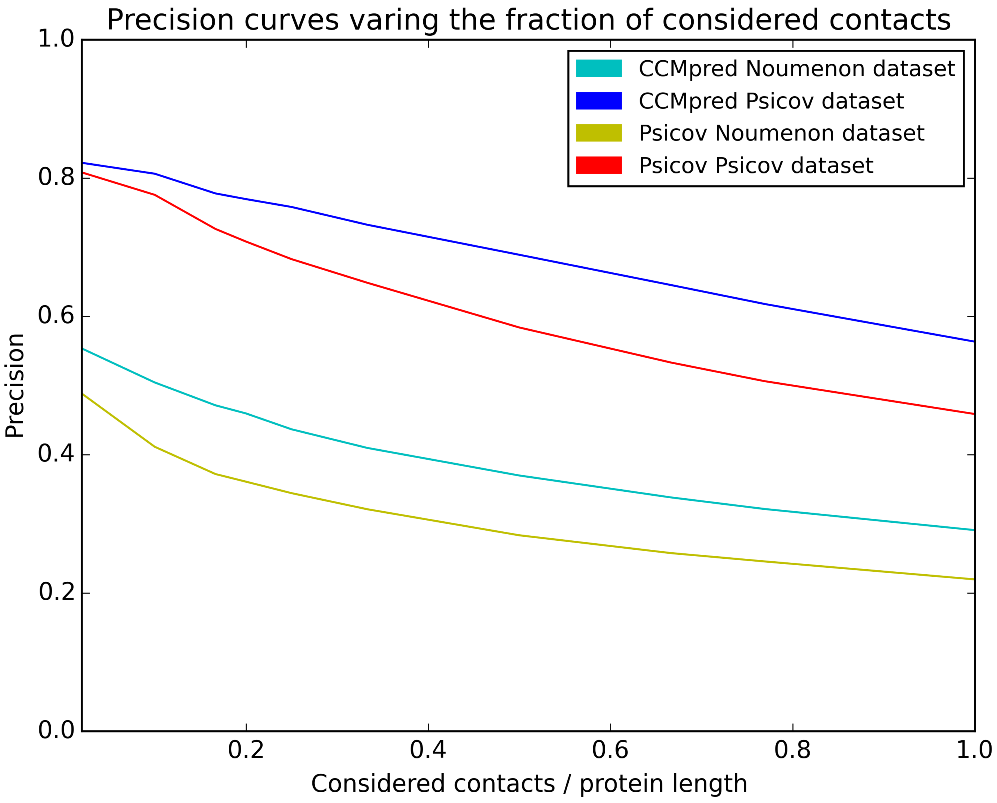

Figure 10: *Precision curves for the PSICOV and CCMpred predictors obtained on the PSICOV and NOUMENON datasets by varying the fraction of contacts considered with respect to the protein length L.*

	NOUMENON best L PPV		PSICOV best L PPV	
Iter	PSICOV	CCMpred	PSICOV	CCMpred
1	0.13	0.18	0.34	0.44
2	0.19	0.26	0.44	0.54
3	0.22	0.29	0.46	0.59

Table 1: *Precision (PPV) scores for the best L predictions on the NOUMENON and PSICOV datasets, computed with PSICOV and CCMpred methods, by varying the number of iterations of Jackhmmer MSAs (E-value=0.0001).*
